# Impact of a mobile health education program on blood pressure and lipid profiles: a cohort study

**DOI:** 10.1186/s12889-026-27656-y

**Published:** 2026-05-08

**Authors:** Jae-Sik Jang, Jaehoon Chung, Kang Hee Kim, Eun Mi Lee, Jong Seon Park, Hwee-Soo Jeong, Joeun Kim, Kyoung-Myoung Chun, Yong-Chol Kwon, Moo-Yong Rhee

**Affiliations:** 1 Division of Cardiology, Department of Internal Medicine, Dongguk University Ilsan Hospital, 27, Dongguk-ro, Ilsandong-gu, Goyang-si, Goyang, Korea; 2 Department of Family Medicine, Dongguk University Ilsan Hospital, Goyang, Korea; 3 Division of Endocrinology and Metabolism, Department of Internal Medicine, Dongguk University Ilsan Hospital, Goyang, Korea; 4 Medical Affairs, VIATRIS Korea, Seoul, Korea

**Keywords:** Hypertension, Dyslipidemia, Digital Health, Mobile Health, Cardiovascular Disease, Awareness, Health Behavior

## Abstract

**Background:**

Hypertension and dyslipidemia are major risk factors for cardiovascular disease, and effective management of these conditions is essential for reducing long-term morbidity. Mobile health (mHealth) education has emerged as a widely used strategy to enhance disease awareness and encourage lifestyle modification. This study evaluated the impact of an mHealth-based education on cardiovascular risk factors and disease awareness among individuals with hypertension.

**Methods:**

This single-arm retrospective cohort study analyzed health screening data collected in 2022 (baseline), 2023 (Phase I), and 2024 (Phase II). Participants received a series of mobile-delivered educational materials focused on hypertension and dyslipidemia, emphasizing lifestyle modification, self-management, and improved understanding of chronic disease risks. Primary outcomes were changes in systolic blood pressure (SBP) and diastolic blood pressure (DBP). Secondary outcomes included changes in triglycerides (TG), low-density lipoprotein (LDL), high-density lipoprotein (HDL), and fasting blood sugar (FBS). Annual prevalence rates of hypertension and dyslipidemia were also assessed.

**Results:**

A total of 408 participants were included in the final analysis. SBP declined from 131 mmHg (IQR: 125–138) at baseline to 130 mmHg (IQR: 124–136) in 2023 and 128 mmHg (IQR: 120–136) in 2024 (*P* < 0.001). DBP decreased from 84 mmHg (IQR: 81–89) to 84 mmHg (IQR: 80–89) in 2023 and 82 mmHg (IQR: 75–89) in 2024 (*P* < 0.001). Hypertension prevalence decreased from 27% to 23.5% and 23%, though changes were not statistically significant. TG levels significantly decreased from 141 mg/dL (IQR: 96–206) to 138 mg/dL (IQR: 91–193) and 127 mg/dL (IQR: 92–185) (*P* < 0.001). LDL showed a statistically significant but small reduction (127 → 128 → 126 mg/dL; *P* = 0.003), while HDL remained unchanged. FBS increased slightly but significantly across the study period (98 → 99 → 99 mg/dL; *P* = 0.01). Dyslipidemia prevalence declined modestly (45.1% → 43.6% → 43.4%) without statistical significance.

**Conclusion:**

This study supports the feasibility of mobile health education as a low-cost strategy for improving blood pressure and lipid control. Strengthening education program content and enhancing participant engagement may further amplify its impact on population cardiovascular health.

**Supplementary Information:**

The online version contains supplementary material available at 10.1186/s12889-026-27656-y.

## Background

Hypertension and dyslipidemia are the most important risk factors for stroke and cardiovascular disease (CVD) [1, 2]. A modest decrease of 5 mmHg in systolic blood pressure (SBP) following a stroke is linked to a more than 20% reduction in the risk of recurrent stroke [2]. However, between 2012 and 2016, the prevalence of ischemic heart disease increased by 13%, while the associated medical expenditures rose by 27% [3]. In recent years, the prevalence has notably increased, particularly among young adults aged 20 to 40 [4, 5]. Despite these risks, awareness of hypertension and dyslipidemia remains low among young adults, and their willingness to actively manage these conditions is often limited [6, 7]. Among young adults, low healthcare utilization rates contribute to delayed diagnosis and a lower prevalence of early detection of hypertension and dyslipidemia. Furthermore, medication adherence remains suboptimal, and lifestyle modifications are often insufficient, leading to overall poor treatment compliance. These factors may exacerbate disease progression and increase the long-term burden of cardiovascular complications [8, 9]. In recent years, the development of digital health technologies utilizing smart devices, including smartphones, smartwatches, and tablets, has significantly advanced, enabling more efficient health monitoring, disease management, and personalized medical interventions [10–12]. Emerging research has shown that digital health technology is a promising tool for managing uncontrolled hypertension, particularly in underserved populations facing barriers to healthcare access [13–15].

This study aims to assess the effectiveness of mobile-based education program in the management of hypertension and dyslipidemia by analyzing health screening data and evaluating their role in enhancing disease awareness.

## Methods

### Study design and participants

**Fig. 1:**
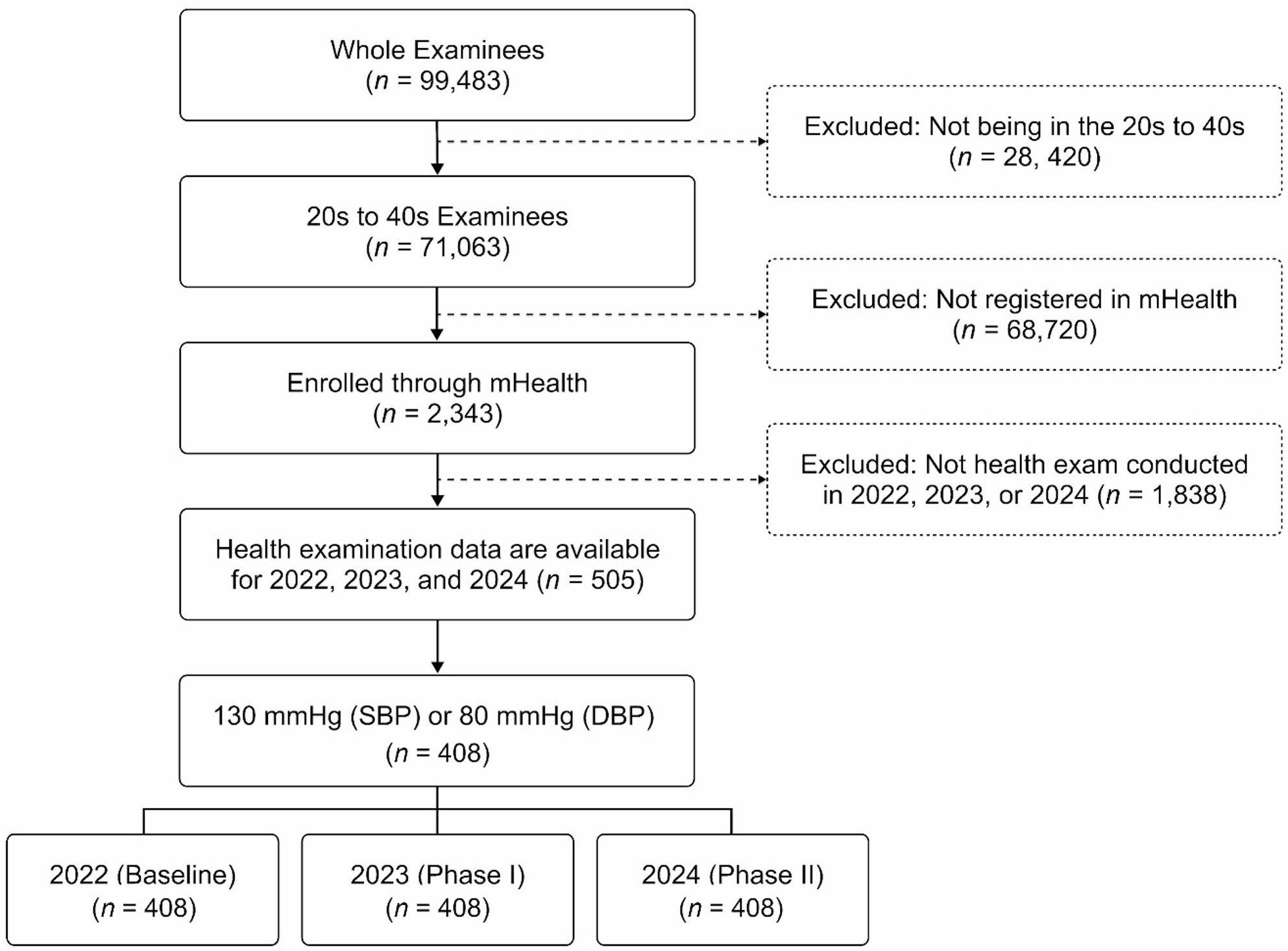
Flow diagram of participants enrollment. Among the individuals participating in the mobile healthcare education program, 408 participants with available health examination data from 2022 to 2024 were included in the analysis

The study population consisted of individuals who underwent health checkups and provided consent for the provision of mobile healthcare content. Eligible participants were adults aged 20–49 years, and those with high normal blood pressure or hypertension (SBP) ≥ 130 mmHg or a diastolic blood pressure (DBP) ≥ 80 mmHg) were included in the analysis, to evaluate whether active lifestyle modification education program delivered via mobile platform could be beneficial (Fig. 1). For the eligible participants, longitudinal changes in blood pressures, lipid profiles and blood sugar were analyzed over a two-year period (2023–2024).

To ensure compliance with ethical guidelines and protect participants’ personal information, all required research-ethics procedures were completed, including the execution of a personal information outsourcing agreement and the administration of a Privacy Assessment Screening Survey (PASS). This retrospective cohort study also received an exemption from the Institutional Review Board of Dongguk University Medical Center (IRB No. DUIH 2025-10-010).

### Content exposure and dissemination

**Fig. 2:**
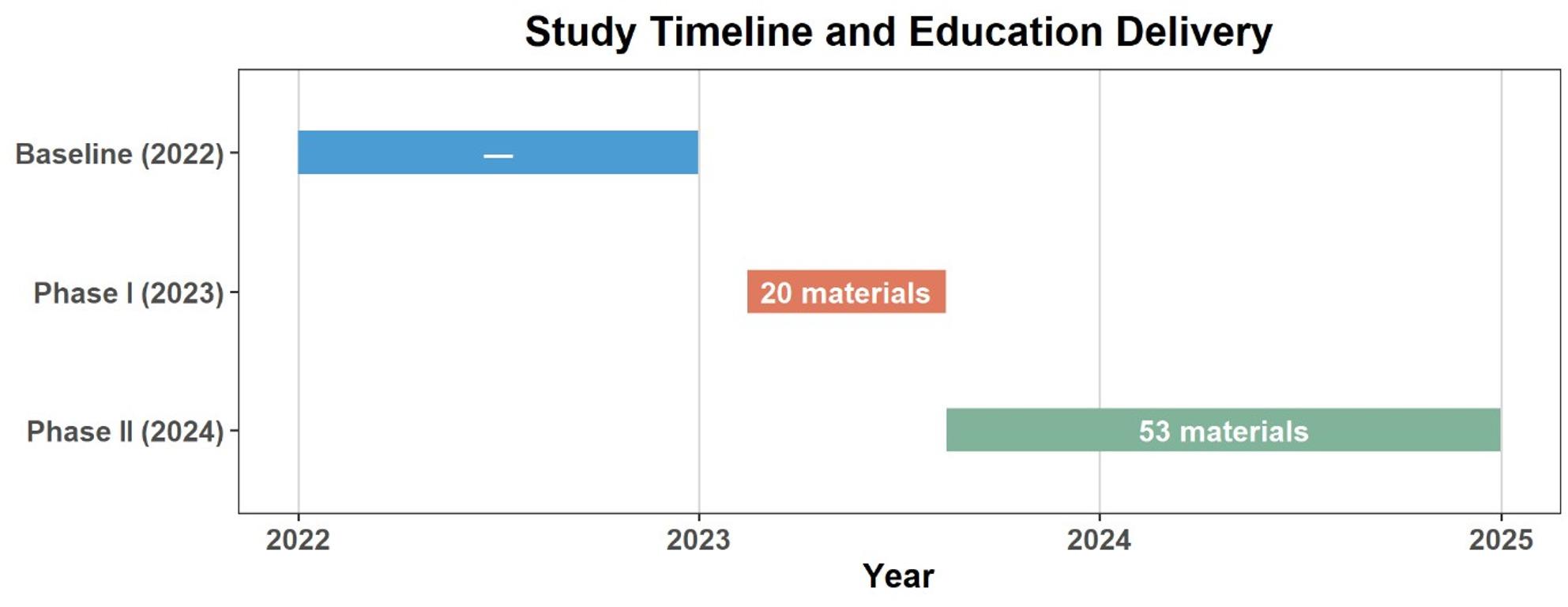
Gantt chart of study timeline and educational content delivery. The Gantt chart summarizes the intervention schedule across three periods: Baseline (2022), Phase I (2023), and Phase II (2024). A total of 20 materials were delivered during Phase I and 53 materials during Phase II, covering hypertension and dyslipidemia education and management resources

Participants were provided with education content on hypertension and dyslipidemia in two phases: Phase I and Phase II. The content covered disease awareness, management, and medication. In Phase I, 20 materials were delivered, whereas in Phase II, 53 materials were provided (Fig. 2). The content was designed to improve awareness of hypertension and dyslipidemia while providing information and tools for their management, ultimately contributing to modest health improvements, including changes in blood pressure (Supplemental Table 1).

**Fig. 3:**
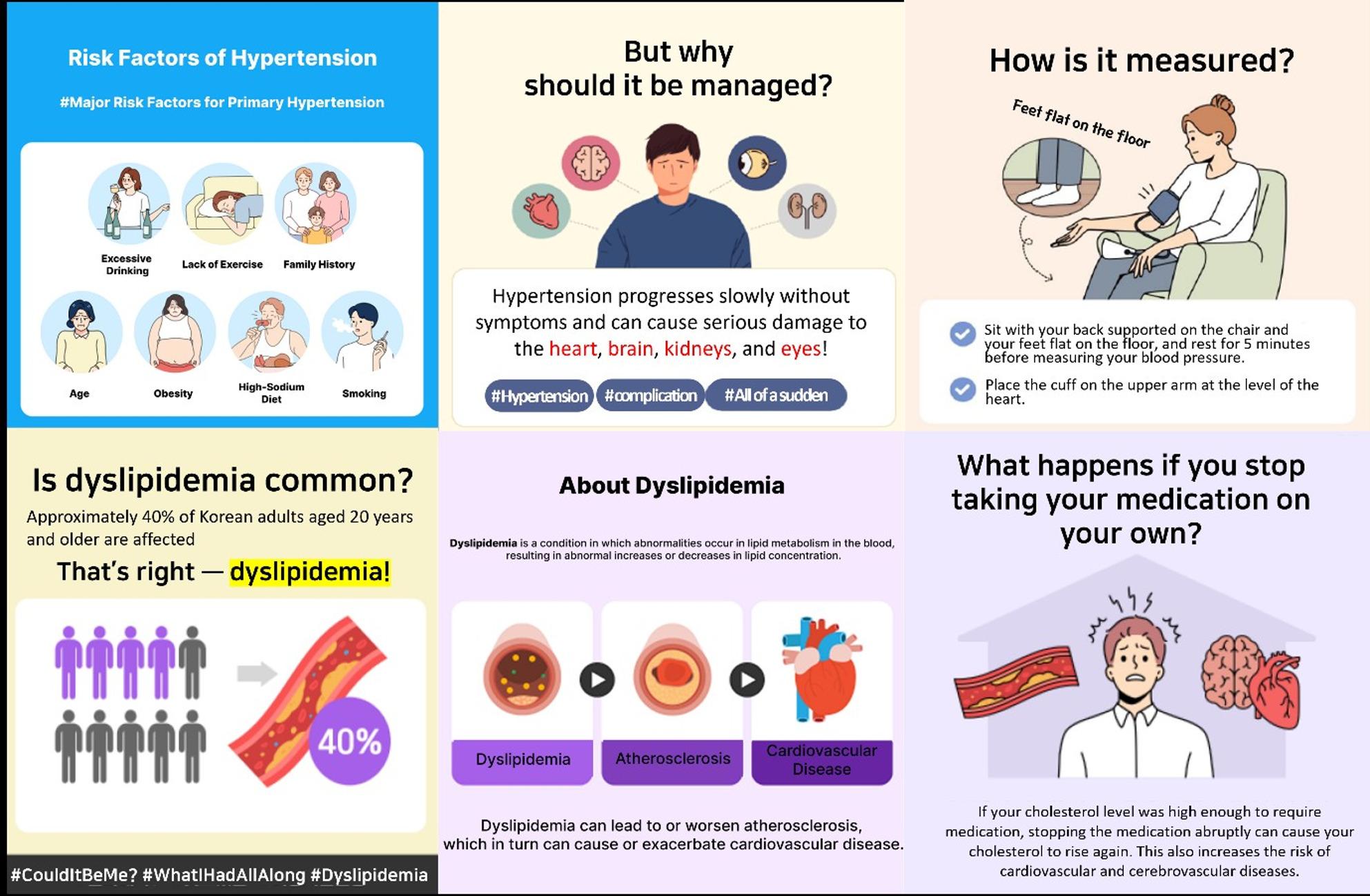
Example of Well-it-go mobile application. Note: The content in this illustration is a translation from Korean to English and may differ slightly from the original expression

The content was first provided through the Kakao Channel Health Log program (Phase I) from February 14 to August 14, 2023, and was later available via the Well-it-go mobile application (Phase II) until December 31, 2024. Through this structured content delivery, participants were encouraged to actively recognize the importance of hypertension and dyslipidemia management and to adopt lifestyle practices supportive of blood pressure and lipid management (Fig. 3).

### Data collection and outcome measures

Health screening data from 2022 served as the baseline for comparison with the 2023 and 2024 health examination data to assess the impact of the education. Among 99,483 individuals who underwent health screening in 2022, 42,756 met the inclusion criteria. Of these, 2,343 consented to participate in the education program, and 408 participants with completed follow-up data were included in the final analysis. The study design focused on evaluating changes across the baseline (2022, pre-education), 2023 (Phase I), and 2024 (Phase II). We investigated the influence of the mobile education on longitudinal changes in SBP, DBP, TG, HDL, and LDL within the participant cohort. Hypertension was defined based on SBP ≥ 140 mmHg or DBP ≥ 90 mmHg [16–18]. Dyslipidemia was defined as triglyceride (TG) levels ≥ 200 mg/dL, high-density lipoprotein cholesterol (HDL-C) levels < 40 mg/dL, or low-density lipoprotein cholesterol (LDL-C) levels ≥ 160 mg/dL [19]. Furthermore, a longitudinal survey was conducted to investigate changes in participants’ awareness of hypertension and dyslipidemia and to assess the impact of mobile health management content (Supplemental Table 2). The survey was made available within the mobile application and could be completed voluntarily by participants. The questionnaire evaluated participants’ awareness and perception of hypertension and dyslipidemia across six key constructs: Disease Perception, Perceived Chronicity, Perceived Controllability, Perceived Treatment Effectiveness, Illness-Related Anxiety, and Disease Awareness. Each construction was assessed with a single item on a 10-point Likert scales (1 = Not at all, 10 = Very much).

### Statistical analysis

Continuous variables were presented as medians with interquartile ranges (IQR), and categorical variables were presented as frequencies with percentages. Comparisons of health data related to hypertension and dyslipidemia across the baseline, Phase I, and Phase II were conducted using the Friedman test, as the assumption of normality was not met in the Shapiro–Wilk test. Post hoc analyses were performed using the Wilcoxon signed-rank test with Bonferroni correction to adjust for multiple comparisons. Because the Friedman test requires complete data for each participant, individuals with missing values in these variables were excluded from the respective analyses. Categorical variables were analyzed using the Chi-squared test. Survey results on participants’ awareness and perception were analyzed using multiple regression analysis. All statistical analyses were performed using R Statistical Software, version 4.2.2 (R Foundation for Statistical Computing, Vienna, Austria).

## Results

### Baseline characteristics

The study participants comprised 345 males (84.6%) and 63 females (15.4%). The median age of the participants in 2022 (Baseline) was 42 years (IQR: 39–47). The prevalence of hypertension was 27% (*n* = 110) and Dyslipidemia was 45.1% (*n* = 184) at baseline (Table 1).

**Table 1:** Baseline characteristics of the study participants

Variables	Baseline (*n* = 408)	*P*-value
Sex		0.002
Male	345 (84.6) ^*^	
Female	63 (15.4)	
Age	42 (39–47)	
Risk Factor		
Hypertension^†^	110 (27)	
Dyslipidemia^‡^	184 (45.1)	

Data are presented as median (interquartile range) or frequencies with percentages unless otherwise indicated. Categorical variables were analyzed using Chi Squared test

^*^A total of 101 male participants had hypertension

^†^Hypertension defined as SBP ≥ 140 mmHg or DBP ≥ 90 mmHg

^‡^Dyslipidemia defined as TG ≥ 200 mg/dL, LDL-C ≥ 160 mg/dL, or HDL-C < 40 mg/dL

### Effects of exposure to mobile health program before and after education

**Fig. 4:**
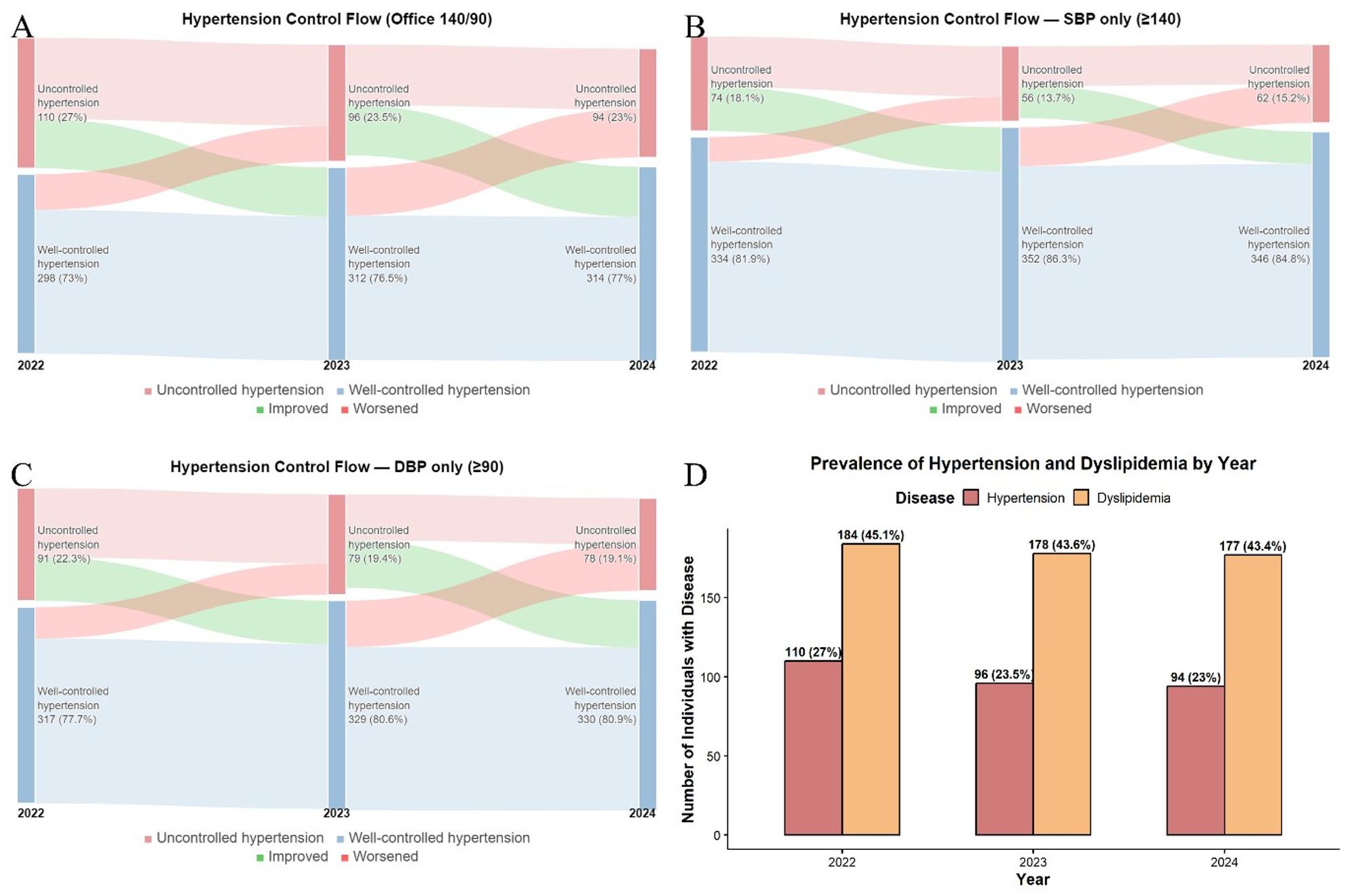
Longitudinal changes in hypertension control and in the prevalence of hypertension and dyslipidemia from 2022 to 2024. **A-C** Alluvial plots illustrating longitudinal transitions in hypertension control status over three consecutive years: **A** control status based on office blood pressure using the 140/90 mmHg criterion, **B** systolic blood pressure (SBP ≥ 140 mmHg), and (C) diastolic blood pressure (DBP ≥ 90 mmHg). Each plot shows transitions between uncontrolled (red) and well-controlled (blue) hypertension, with flow width representing the proportion of participants moving between categories. **D** Yearly prevalence of hypertension and dyslipidemia presented as bar charts. Values above each bar indicate the number of individuals with the corresponding condition and the proportion relative to all participants assessed in that year. Both conditions showed modest reductions in prevalence over time following implementation of the mobile-based lifestyle modification program

Analysis of hypertension-related indicators showed a significant decrease in median SBP, from 131 mmHg (IQR: 125–138) in 2022 (Baseline) to 130 mmHg (IQR: 124–136) in 2023 (Phase I), followed by a further decline to 128 mmHg (IQR: 120–136) in 2024 (Phase II), as shown in Table 2 (*P* < 0.001). Median DBP also decreased from 84 mmHg (IQR: 81–89) in baseline to 84 mmHg (IQR: 80–89) in 2023, followed by a further decline to 82 mmHg (IQR: 75–89) in 2024, showing a statistically significant reduction (*P* < 0.001) (Table 2). The prevalence of hypertension, decreased from 27.0% (*n* = 110) at baseline to 23.5% (*n* = 98) in 2023 and 23.0% (*n* = 94) in 2024, although the reductions were not statistically significant (Fig. 4).

**Table 2:** Comparison of Health Data Across 2022, 2023, and 2024

Variable	2022 (*n* = 408)	2023 (*n* = 408)	2024 (*n* = 408)	*P*-value
TG (mg/dL)	141 (96–206) ^‡§^	138 (91–193) ^†^	127 (92–185) ^†^	< 0.001^*^
HDL (mg/dL)	43 (49–58)	43 (50–58)	50 (43–59)	0.15
LDL (mg/dL)	127 (104–150) ^‡§^	128 (101–151) ^†^	126 (103–153) ^†^	0.003^*^
SBP (mmHg)	131 (125–138) ^‡§^	130 (124–136) ^†^	128 (120–136) ^†^	< 0.001^*^
DBP (mmHg)	84 (81–89) ^‡§^	84 (80–89) ^†§^	82 (75–89) ^†‡^	< 0.001^*^
FBS (mg/dL)	98 (91–106) ^§^	99 (92–106)	99 (92–108) ^†^	0.01^*^

Data are presented as median (interquartile range) unless otherwise indicated. Continuous variables were compared using Friedman test according to the results of the Shapiro–Wilk normality test. Continuous variables were compared using the Friedman test according to the results of the Shapiro–Wilk normality test

*TG*  triglycerides, *HDL  *high–density lipoprotein, *LDL*  low-density lipoprotein, *SBP  *systolic blood pressure, *DBP  *diastolic blood pressure, *FBS  *fasting blood sugar

*P < 0.05 for comparison among 2022, 2023, and 2024

^†^ Indicates statistically significant with 2022

^‡^ Indicates statistically significant with 2023

^§^ Indicates statistically significant with 2024

Although a slight increase was observed in 2023, median TG levels showed an overall downward trend, decreasing from 141 mg/dL (IQR: 96–206) in 2022 to 138 mg/dL (IQR: 91–193) in 2023, and then declining to 127 mg/dL (IQR: 92–185) in 2024 (*P* < 0.001) (Table 2). Median LDL levels showed a slight but statistically significant decrease, from 127 mg/dL (IQR: 104–150) at baseline to 126 mg/dL (IQR: 103–153) in 2024 (*P* = 0.003). However, no significant changes were observed in HDL. The prevalence of dyslipidemia showed a slight decline, from 45.1% (*n* = 184) at baseline to 43.6% (*n* = 178) in 2023 and 43.4% (*n* = 177) in 2024 (Fig. 4D).

In contrast, the median fasting blood sugar (FBS), a diabetes-related indicator, showed a slight but statistically significant increase, rising from 98 mg/dL (IQR: 91–106) in 2022 and 99 mg/dL (IQR: 92–106) in 2023 to 99 mg/dL (IQR: 92–108) in 2024 (*P* = 0.01).

Multiple regression analysis showed a slight upward trend in awareness and perception, but none were statistically significant. Perceived Controllability showed a marginal increase (*P* = 0.083), while all other constructions remained stable. The models had low explanatory power (R² < 0.01), indicating minimal impact of time on perception changes (Supplemental Table 3).

## Discussion

This two-year cohort study demonstrated that a mobile health (mHealth) education program was associated with modest yet statistically significant improvements in blood pressure and lipid profiles among adults with high-normal blood pressure or hypertension. Specifically, systolic and diastolic blood pressure decreased by approximately 2–3 mmHg, accompanied by slight reductions in triglyceride and LDL cholesterol levels. Importantly, the benefits became more apparent after two consecutive years of exposure, suggesting that sustained participation and cumulative engagement are critical to achieving measurable cardiometabolic improvements. Although the prevalence of hypertension and dyslipidemia showed numerical declines over time, these changes were not statistically significant and should therefore be interpreted cautiously. These patterns may suggest a potential favorable trend, but definitive conclusions regarding effectiveness cannot be drawn.

### Comparison with previous studies

Our findings are consistent with prior meta-analyses of mHealth education that reported 2–5 mmHg reductions in systolic blood pressure through smartphone-based education, telemonitoring, or text message support [13–15, 20, 21]. However, most of these trials enrolled middle-aged or elderly adults with established hypertension, whereas the present study targeted a younger population (20–49 years). This distinction is important, as hypertension and dyslipidemia awareness in young adults remains low despite a rising prevalence [4, 5]. The positive results in this cohort highlight the feasibility and potential of mobile-based education program to engage individuals who rarely seek in-person healthcare but are highly receptive to digital communication. However, even small reductions in systolic blood pressure at the population level have been associated with meaningful reductions in cardiovascular risk in prior epidemiologic studies demonstrating a continuous relationship between blood pressure and cardiovascular risk [22, 23].

The observed lipid improvements were slight but clinically favorable. The decline in triglycerides and LDL aligns with previous digital education programs emphasizing self-monitoring and dietary feedback [21, 24]. These effects likely reflect incremental behavioral modifications—improved meal patterns, reduced alcohol consumption, and increased physical activity.

### Possible mechanisms and behavioral pathways

The physiological improvements observed may be explained through behavioral reinforcement mechanisms. Regular exposure to disease-related messages, visual cues, and simplified health information likely enhanced risk perception and self-efficacy. In behavioral economics terms, this reflects a “nudge” effect—small, context-sensitive prompts that guide individuals toward healthier choices without explicit enforcement [25]. Over time, such micro-interventions may lead to meaningful changes in daily habits, including reduced salt intake, weight control, and adherence to physical activity routines [26].

Moreover, mobile health education programs may help address several practical barriers that can hinder primary prevention—such as limited time, transportation demands, and the perceived burden of clinic visits—by providing flexible, low-effort, and asynchronous engagement that can be more easily incorporated into daily life. The cumulative exposure observed in this trial, which included more diverse and personalized content, likely amplified these benefits and underscores the need for dynamic rather than static health education approaches.

### Unexpected findings: increase in fasting blood glucose

An unexpected finding was the small but statistically significant rise in fasting blood glucose. Several explanations are possible. First, the program’s educational focus on hypertension and lipid control may have inadvertently neglected balanced guidance on glucose regulation, particularly regarding carbohydrate quality and meal timing. Second, the observed increase in fasting blood glucose cannot be clearly attributed to specific behavioral or pharmacologic changes because data on diet, body weight, and medication adjustments were not collected; therefore, potential explanations such as an imbalance in education program content or selective participation of higher risk individuals should be regarded as speculative and interpreted with caution. Future versions of the program should include broader metabolic education—such as weight management, blood sugar control, and healthy sleep habits. Expanding the focus beyond blood pressure and lipid management will help support more balanced overall metabolic health.

### Public health and policy implications

Even modest reductions in systolic blood pressure and LDL cholesterol could contribute to reductions in cardiovascular mortality at the population level, as suggested by large-scale epidemiologic and modeling studies [8, 27]. Given the high penetration of smartphones in Korea and many other countries, low-cost digital education programs represent a scalable tool that can support blood pressure control and promote overall cardiovascular health, thereby complementing national health screening initiatives [28]. Integrating automated digital counseling or personalized “health feedback loops” after routine screenings may enhance awareness of hypertension, promote physical activity, and improve treatment adherence, thereby supporting better overall disease management [8, 29]. Importantly, mHealth education can reach busy younger adults and working populations with limited access to primary care, supporting earlier recognition and management of hypertension and dyslipidemia.

As highlighted in the WHO report, digital health education—including mobile health, telemedicine, and chatbots—could be a promising public health strategy for improving the management of hypertension, diabetes, and cardiovascular diseases at very low cost [30]. These digital approaches provide real-time behavior-change content, support self-management, and help overcome barriers related to distance, transportation, and cost, thus improving continuity of care. The WHO also notes that even relatively small investments in digital health can yield benefits that far exceed their costs, underscoring the scalability and value of digital solutions for the prevention and control of non-communicable diseases (NCDs). Accordingly, WHO encourages governments to incorporate national digital health strategies into NCD prevention and management frameworks.

### Limitations

Several limitations warrant consideration. First, this study was designed in a single arm without a control group. Therefore, it is unclear whether the improvements in blood pressure and dyslipidemia-related metrics were directly attributable to exposure to mobile healthcare content. In addition, the observed changes may partly reflect secular trends or regression to the mean rather than the intervention itself. No information on medication use limits the analysis for causal inference from them, and confounding effects cannot be fully excluded. Additionally, information on other potential confounders such as dietary habits, physical activity, and socioeconomic or educational status was not available, which may further limit interpretation of the findings. Future studies should collect these variables to allow for more comprehensive multivariable analyses. Because engagement metrics (e.g., frequency and duration of content exposure) were not captured, dose–response relationships could not be evaluated. In addition, the perception survey had limited and inconsistent response rates across waves, reducing the ability to detect significant changes in awareness. Finally, the study population, primarily male employees in their forties, may not fully represent the broader community, limiting generalizability.

## Conclusion

This study supports the feasibility of mobile health education as a sustainable, low-cost strategy to improve blood pressure and lipid control in adults. Strengthening education program content, integrating engagement analytics, and aligning with national digital health policy could magnify the impact of such education on population cardiovascular health.

## Supplementary Information

Supplementary Material 1.

## Data Availability

The datasets used and analyzed during the current study are available from the corresponding author on reasonable request.

## Abbreviations

SBP: Systolic blood pressure
DBP: Diastolic blood pressure
TG: Triglycerides
LDL: Low-density lipoprotein
HDL: High-density lipoprotein
FBS: Fasting blood sugar
