# Supplementary material for: Impact of a mobile health education program on blood pressure and lipid profiles: a cohort study
Source: BMC Public Health. 2026 May 8;26:1994. doi: 10.1186/s12889-026-27656-y (PMC13321482; doi:10.1186/s12889-026-27656-y)
Supplement: Supplementary file 1 — Supplementary Material 1. [file 12889_2026_27656_MOESM1_ESM.docx]

**Supplementary material**

**Supplemental Table 1.** Overview of mobile health program content in Phase I and Phase II.

| NO | Phase I | Phase II |
| --- | --- | --- |
| 1 | Hypertension, the silent killer. | What Is Hypertension? |
| 2 | Dyslipidemia: Why Is Management Important? | Hypertension — the Silent Killer |
| 3 | Should I See a Doctor for Hypertension? | Rising Hypertension in Young Adults |
| 4 | How Dyslipidemia Causes Disease | How to Measure Blood Pressure Correctly |
| 5 | Hypertension Is Getting Younger. | Hypertension and the Brain |
| 6 | Do I Need Medicine for Dyslipidemia? | Hypertension and the Heart |
| 7 | Do I Need Medicine for High Blood Pressure? | Hypertension and the Kidneys |
| 8 | Know Your Heart Disease Risks! | Hypertension and the Eyes |
| 9 | Stay Consistent — Control Your Blood Pressure! | Hypertension Treatment |
| 10 | Smart Tips to Manage Dyslipidemia | Hypertension and Complications |
| 11 | Eat Smart for Healthy Blood Pressure | Hypertension Risks 10 Years Later |
| 12 | Protect Your Brain from Stroke | Personalized Hypertension Treatment Plan |
| 13 | Your Personal Hypertension Plan | 7 Golden Rules for Blood Pressure Control |
| 14 | Stick to Your Plan — Manage Dyslipidemia Right | Am I Hypertensive? |
| 15 | Move More, Control Your Blood Pressure | Should I Visit a Doctor for Hypertension? |
| 16 | Your Personal Dyslipidemia Plan | Should I Take Medicine for Hypertension? |
| 17 | 7 Golden Rules for Blood Pressure Control | Why Consistent Medication and Follow-Up Matter |
| 18 | Young but Not Immune — Watch Your Blood Pressure! | Tips for Consistent Medication Adherence |
| 19 | Stay on Track with Your Medication | Healthy Eating for Blood Pressure Control |
| 20 | 5 Habits for a Healthy Blood Pressure | Exercise for Healthy Blood Pressure |
| 21 | N/A | What Is Dyslipidemia? |
| 22 | N/A | Even Young People Need to Manage Dyslipidemia |
| 23 | N/A | What Is Hyperlipidemia? |
| 24 | N/A | Why Is Dyslipidemia Management Important? |
| 25 | N/A | 5 Habits for Healthy Blood Pressure |
| 26 | N/A | Personalized Dyslipidemia Treatment Plan |
| 27 | N/A | How Dyslipidemia Causes Disease |
| 28 | N/A | What Is Cardiovascular Disease? |
| 29 | N/A | What Is Cerebrovascular Disease? |
| 30 | N/A | Should I Visit a Doctor for Dyslipidemia? |
| 31 | N/A | Dyslipidemia Treatment |
| 32 | N/A | Should I Take Medicine for Dyslipidemia? |
| 33 | N/A | Smart Ways to Manage Dyslipidemia |
| 34 | N/A | Why Consistent Medication and Follow-Up Are  Important in Dyslipidemia Management |
| 35 | N/A | Healthy Eating Habits for Dyslipidemia Management |
| 36 | N/A | Exercise for Dyslipidemia Control |
| 37 | N/A | DASH Diet |
| 38 | N/A | Mindful Eating |
| 39 | N/A | Understanding Nutritional Labels |
| 40 | N/A | Eating Out the Healthy Way |
| 41 | N/A | Choosing Healthy Snacks |
| 42 | N/A | Reducing Salt Intake |
| 43 | N/A | Increasing Daily Activity |
| 44 | N/A | Managing Stress |
| 45 | N/A | Breathing Exercises |
| 46 | N/A | 478 Breathing Technique |
| 47 | N/A | Drinking Water Properly |
| 48 | N/A | Lose Fat, Gain Health |
| 49 | N/A | Why Weight Control Matters for Diabetics |
| 50 | N/A | How to Calculate Your BMI |
| 51 | N/A | First Step to a Healthy Lifestyle |
| 52 | N/A | Dietary Tips to Lower Cholesterol |
| 53 | N/A | Regular Health Check-Ups |

Note.— This table presents a Korean-to-English translation, and some expressions may not fully reflect the original wording.

**Supplemental Table 2.** Contents satisfaction Survey

| No. | Question | Responses |
| --- | --- | --- |
| 1 | How satisfied are you with the content? | 1–5 scale |
| 2 | Which aspect of managing your hypertension (or dyslipidemia) are you currently most concerned about? | Regular check-ups; Lifestyle management; Medication adherence; Not managing at all |
| 3 | Through the content provided, which area of chronic disease management has become more important to you compared with before? | 1–5 scale |
| 4 | Through the content provided, which area of chronic disease management have you practiced more actively than before? | 1–5 scale |
| 5 | When consulting with a doctor or nurse, do you provide all the information necessary for your care? | Almost never; Occasionally; Frequently |
| 6 | When consulting with a doctor or nurse, do you ask appropriate questions? | Almost never; Occasionally; Frequently |
| 7 | When consulting with a doctor or nurse, do you ask again if there is anything you do not understand? | Almost never; Occasionally; Frequently |
| 8 | Do you prefer to learn as much as possible about your health? | Almost never; Occasionally; Frequently |
| 9 | Do you actively seek and trust health information relevant to your condition? | Almost never; Occasionally; Frequently |
| 10 | How much do you think your hypertension (or dyslipidemia) affects your daily life? | 0–10 scale |
| 11 | How long do you think your hypertension (or dyslipidemia) will persist? | 0–10 scale |
| 12 | How well do you think your hypertension (or dyslipidemia) can be controlled? | 0–10 scale |
| 13 | How helpful do you think treatment will be for your hypertension (or dyslipidemia)? | 0–10 scale |
| 14 | How often do you experience symptoms related to your hypertension (or dyslipidemia)? | 0–10 scale |
| 15 | How worried are you about your hypertension (or dyslipidemia)? | 0–10 scale |
| 16 | How well do you understand your hypertension (or dyslipidemia)? | 0–10 scale |
| 17 | To what extent do you think your emotions influence your hypertension (or dyslipidemia)? | 0–10 scale |
| 18 | Choose the three factors you believe most contributed to the development of your hypertension (or dyslipidemia). | Natural factors; Occupational factors; Physical changes; Psychological factors; Environmental factors |

Note.— This table presents a Korean-to-English translation, and some expressions may not fully reflect the original wording.

**Supplementary Table 3.** Multiple Regression Analysis Results for subgroup analysis (survey data)

| Variable | Disease Perception | Perceived Chronicity | Perceived Control Ability | Perceived Treatment Effectiveness | Illness-Related Anxiety | Disease Awareness |
| --- | --- | --- | --- | --- | --- | --- |
| Estimate (SE) | 0.187 (0.201) | 0.040 (0.192) | 0.274^*^ (0.158) | 0.148 (0.153) | 0.082 (0.196) | 0.217 (0.177) |
| Observations | 286 | 286 | 286 | 286 | 286 | 286 |
| R^2^ | 0.003 | 0.0002 | 0.011 | 0.003 | 0.001 | 0.006 |

Note—.*P < 0.1; **P < 0.05; ***P < 0.01
